# Regulation of oestrogen signalling by the receptor tyrosine kinase cKIT stabilises Barrett's oesophagus pre-malignant state

**DOI:** 10.1242/bio.062802

**Published:** 2026-09-08

**Authors:** Kin Man Suen, Safoura Zahed Mohajerani, Poppy G. Tyrer, Henry W. Clark, Christopher M. Jones, John E. Ladbury

**Affiliations:** ^1^ Faculty of Biological Sciences, University of Leeds, Leeds LJ2 9JT, UK; ^2^ Leeds Institute of Rheumatic and Musculoskeletal Medicine, School of Medicine, Worsley Building, University of Leeds, Leeds LS2 9JT, UK; ^3^ Early Cancer Institute, University of Cambridge, Cambridge CB2 0XZ, UK; ^4^ Addenbrooke's Hospital, Cambridge University Hospitals NHS Foundation Trust, Cambridge CB2 0QQ, UK

**Keywords:** Oesophageal adenocarcinoma, High-grade dysplasia, Tight junction proteins, Occludin, Oestrogen receptors

## Abstract

The pre-malignant precursor to oesophageal adenocarcinoma (OAC), Barrett's oesophagus (BO), is prevalent in 10-15% of patients with chronic gastroesophageal acid reflux. However, in most cases, the BO condition is stable to cancer promotion; i.e. less than 1% of patients develop OAC. Here, we investigate whether there are factors that can act to prevent BO patients from developing OAC. The receptor tyrosine kinase, cKIT, is expressed in Barrett's tissue, but sparingly so in either normal or OAC tissue. To investigate whether cKIT influences cellular function that might be consistent with sustaining BO, we knocked out the *KIT* gene. Gene Ontology analysis revealed that cells deleted for cKIT showed differentially expressed genes (DEGs) affecting functions associated with intercellular interactions and tight junction formation. Further analysis of DEGs showed that the absence of cKIT, as well as treatment with acid and bile salts, affected genes associated with oestrogen signalling. Under these conditions, the tight junction protein Occludin was found to translocate to the nucleus. Thus, the cKIT receptor, through regulating intracellular oestrogen signalling, affects cellular tight junction formation. Reducing cKIT function in BO tissue potentially leads to elevated permeability and exposure of basal and stromal cells to luminal noxious agents which could act as OAC promoters.

## Teaser

**Summary:** The receptor tyrosine kinase cKIT is expressed at an elevated level in Barrett's oesophagus compared to normal and oesophageal adenocarcinoma tissues. By regulating oestrogen signalling, it can limit the promotion of cancer.

## INTRODUCTION

There is an increasing focus on the factors promoting progression of premalignancy to cancer (Hill et al., 2024). This includes Barrett's oesophagus (BO), which is the precursor for oesophageal adenocarcinoma (OAC). The genetic drivers of OAC that develop in BO patients have been investigated (Zamani et al., 2026); however, little attention has been paid to factors that may prevent the promotion of OAC, i.e. stabilise the BO condition. BO is detected in 10-15% of patients with chronic gastroesophageal reflux disease (GORD; Shaheen et al., 2016) and has a significantly higher occurrence rate in men and postmenopausal women (Asanuma et al., 2016; Dong et al., 2020; Melquist et al., 2022; Beaton et al., 2024). BO develops as a mosaic of intestinal- and gastric-like epithelium that replaces the oesophageal normal squamous epithelium proximal to the gastroesophageal junction. This occurs as an adaptation to exposure to injurious gastrointestinal refluxate but predisposes to the development of OAC (Hayakawa et al., 2021; Killcoyne and Fitzgerald, 2021). Pathologically, BO is usually characterised as one of three stages: non-dysplastic, low-grade dysplasia (LGD) and high-grade dysplasia (HGD).

Although the number of patients presenting with pre-malignant LGD BO who progress to intramucosal adenocarcinoma is low (<1%), HGD elevates the risk of progression to >20% (Hvid-Jensen et al., 2011). Progression through the dysplastic stages is accompanied by changes to genetic and non-genetic features of the tissue cells (Zamani et al., 2026). Despite BO and OAC being associated with high mutational burden (see TCGA data: https://www.cancer.gov/ccg/research/genome-sequencing/tcga/using-tcga-data/citing), the number of recurrent point mutations in cells is low, inferring that the impact of driver mutations is outweighed by changes in gene transcription levels and protein expression.

The factors that contribute to maintaining the BO lesion or preventing promotion to OAC in these patients are uncertain. The low levels of progression and low risk to survival of patients suggest that cells within this tissue have features that make them robust to frequent episodes of acid/bile salt reflux over the patients’ lifetime. Progression to HGD, and ultimately OAC, is accompanied by the gradual erosion of these features, accompanied by accumulation of genetic and epigenetic alterations leading to promotion of the cancer phenotype (Zamani et al., 2026). The factors that contribute to stabilisation (i.e. prevent OAC promotion) within BO lesion in these patients are uncertain. Given that the proteome is markedly less studied in BO, we interrogated a prior meta-analysis of patient data (Maag et al., 2017; Jones, 2021; Fig. S1) in order to identify protein signalling pathways that could be hallmarks of BO stabilisation. This highlighted receptor tyrosine kinase (RTK) expression profiles that showed differential expression in HGD BO, normal squamous epithelium and OAC. Amongst these, the cKIT receptor is expressed in BO cells but only marginally expressed in normal and cancer cells. This suggested that cKIT may feature in sustaining the BO state, thereby preventing or delaying progression to OAC.

Plasma-membrane-localised cKIT is stimulated by the extracellular stem cell factor (SCF) ligand. The *KIT* gene is found on chromosome 4q12 (Douville et al., 2021). Abnormalities and instability of this chromosome are associated with BO progression where hyperploidy is common. The role of cKIT has largely been reported in early activation, expansion and differentiation of hepatic oval stem cells (Fujio et al., 1994), early haematopoietic cells, mast cells, and dendritic cells (Metcalfe et al., 1997; Ishii et al., 2005; Ray et al., 2010). SCF and cKIT are additionally important in early response to acute liver failure (Nassar et al., 2009; Shaker, 2014) and liver regeneration post-transplantation (Li et al., 2013). The receptor's activity has also been associated with gastrointestinal tissue in studies on inflamed intestinal crypts (Dahlen et al., 2001; Dubreuil et al., 2009) and phosphoinositide 3 kinase (PI3K)/AKT signalling in organoid models of mouse intestinal crypts (Schmitt et al., 2018).

Here, we investigate the role of cKIT in BO with a particular interest in its function in impeding the promotion of OAC. Through adopting a cell line representing dysplastic progression in BO and exposing it to acid and bile salts [bile acid (BA)] to attempt to mimic acid reflux, we characterised molecular features associated with stabilising Barrett's state. Knocking out the *KIT* gene had a significant impact on the expression of genes associated with oestrogen signalling in the presence of BA, thus providing a link to BO via the known prevalence in male and postmenopausal female patients. Further investigation through Gene Ontology (GO) revealed that signalling via this hormone regulated the function of a subset of proteins associated with tight junction formation. Downstream effects of deleting cKIT cellular function revealed the aberrant behaviour of the tight junction protein (TJP) Occludin, implicating a potential increase in tissue permeability and the exposure of basal and stromal tissues to acid and bile salt stress. We therefore propose that the presence of cKIT in BO, through regulation of oestrogen signalling, stabilises pre-malignant oesophageal lining and hence could provide opportunities for therapeutic intervention to prevent progression to malignancy.

## RESULTS

### Tissue integrity function in BO oesophageal cells

**Fig. 1.**
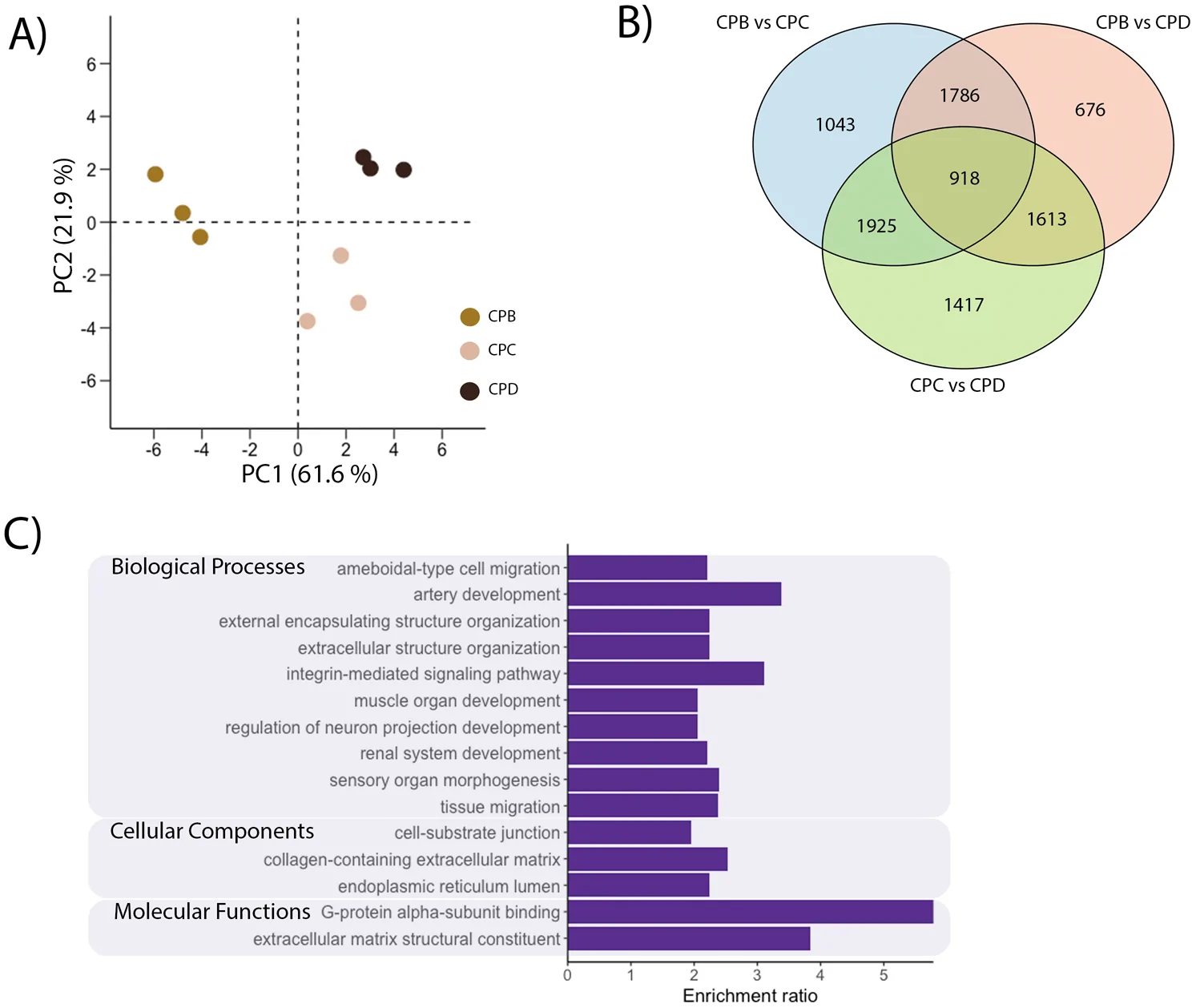
**Comparisons between the dysplasticBOcell lines CPB, CPC and CPD.** (A) PCA shows CPC and CPD are more similar to each other compared to CPB. (B) Overlapping DEGs in pairwise comparisons of CPB versus CPC, CPB versus CPD, and CPC versus CPD (*P*_adj_<0.05). (C) GO term analysis of the 918 DEGs shared across all three pairwise comparisons between the CPB, CPC and CPD cell lines. Enriched terms (FDR<0.01) are grouped by the GO domain (Biological Processes, Cellular Components, Molecular Functions).

For the purposes of this study, we initially adopted cell lines derived from patients presenting different characteristics of HGD BO: CPB (CP-52731, BAR-10T), CPC (CP-94251) and CPD (CP-18821) (Palanca-Wessels et al., 1998; Kosoff et al., 2012). Their protein-coding transcriptomes were sequenced, and data were analysed via principal component analysis (PCA) (Fig. 1A). The CPC and CPD cell lines appear more similar to each other than to CPB, with CPC and CPB being the most different from each other, consistent with previous pathological characterisation (Kosoff et al., 2012). When comparing CPB and CPC, we found 5672 differentially expressed genes (DEGs); comparing CBP and CPD, we found 4493 DEGs; comparing CPC and CPD, we found 5873 DEGs (Fig. 1B). Taking the DEGs for each pair of cell types, 918 of these DEGs overlap in all three comparisons, suggesting that this set of genes distinguishes all three cell lines. The role of these DEGs was investigated using GO term analysis (Fig. 1C; Fig. S2). Genes that differentiate all three cell lines include several terms that invoke functions that contribute to intercellular contact and tissue integrity, such as cell-substrate junctions, integrin-mediated signalling pathways and extracellular structure organisation.

### Impact of cKIT knockout on the function of Barrett's cells

**Fig. 2.**
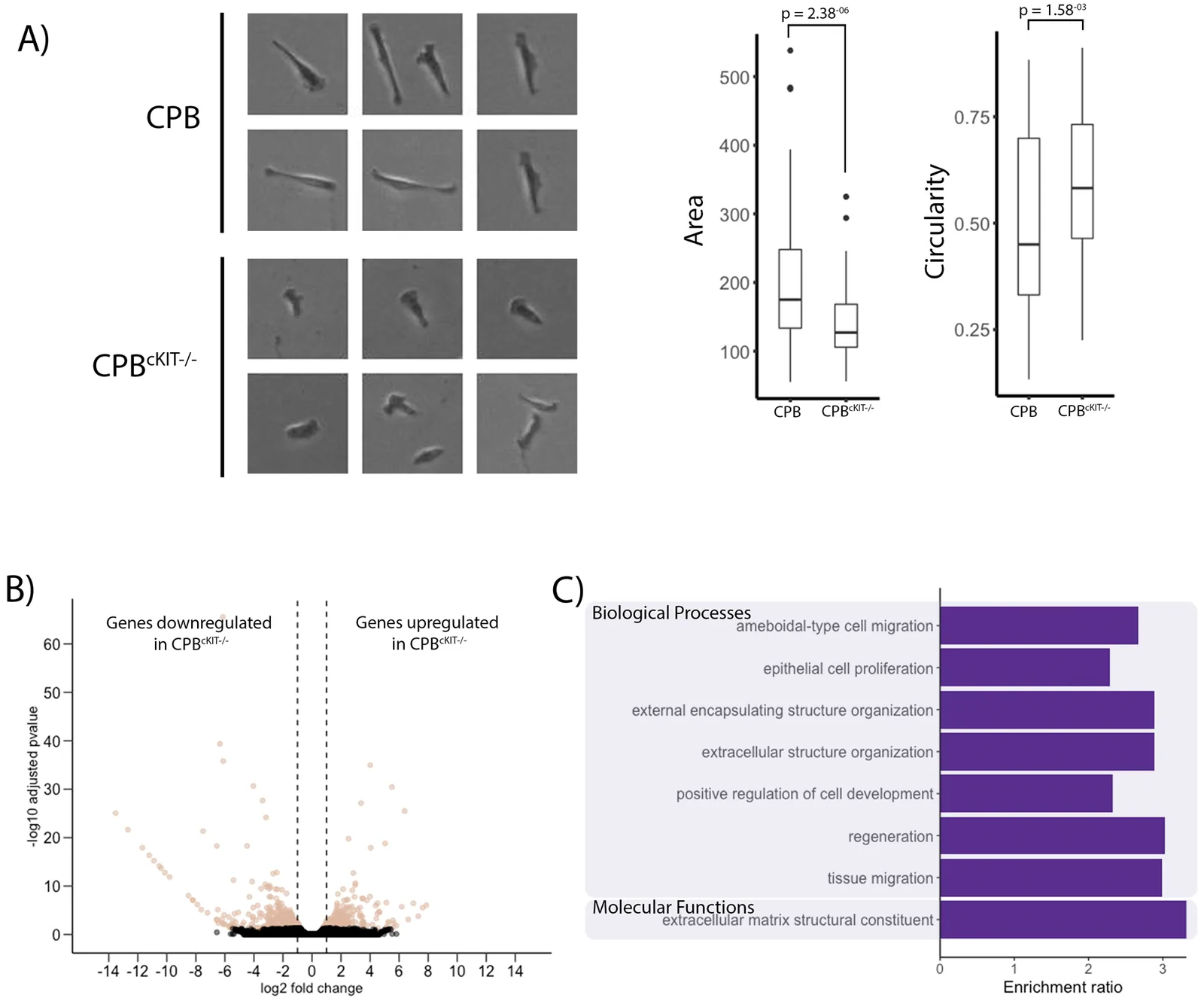
**cKIT ablation in CPB leads to morphological and gene expression changes.** (A) Left panel: Representative of CPB and CPB^cKIT−/−^ cells stained with Crystal Violet. Right panel: CPB^cKIT−/−^ cells are smaller and more circular than CPB cells (*n*=80 in each line). (B) Volcano plot comparing the transcriptome of CPB and CPB^cKIT−/−^. DEGs (*P*_adj_<0.05) are in pink. (C) GO term analysis on DEGs in CPB^cKIT−/−^ compared with CPB. Enriched terms (FDR<0.01) are grouped by the GO domain (Biological Processes and Molecular Functions); no terms in the Cellular Components domain passed the threshold.

Based on previously reported meta-analysis of patient data (Maag et al., 2017; Jones, 2021), we were able to examine the role of all known RTKs in BO by comparison with data from normal and OAC tissues. Amongst all the RTKs reported, cKIT showed low overall expression (Fig. S1). However, unlike the other RTKs, cKIT showed elevated expression only in HGD Barrett's tissue, suggesting that it could be responsible for downstream signalling associated with sustaining the BO condition. Based on its usage to represent HGD in previously published studies (e.g. Bhardwaj et al., 2016; Bhat et al., 2018), we selected the CPB cell line for further experimentation. We produced a CPB cell line in which CRISPR was used to knock out the *KIT* gene. The cells from the knockout cell line (CPB^cKIT−/−^; Fig. S3) were generally smaller and more circular in 2D culture (Fig. 2A). RNA sequencing (RNA-seq) analysis performed on CPB^cKIT−/−^ revealed a large number of DEGs when compared to CPB parental cells; 346 and 218 genes were downregulated and upregulated, respectively, when cKIT was knocked out (Fig. 2B). GO term analysis (Fig. 2C) reveals that cKIT transcription-related signalling plays a prominent role in extracellular structure organisation and cell migration. The GO term ‘regeneration’ is also enriched, which fits with the function that cKIT adopts in intestinal stem cells and liver repair (Fujio et al., 1994; Wang et al., 2021).

**Fig. 3.**
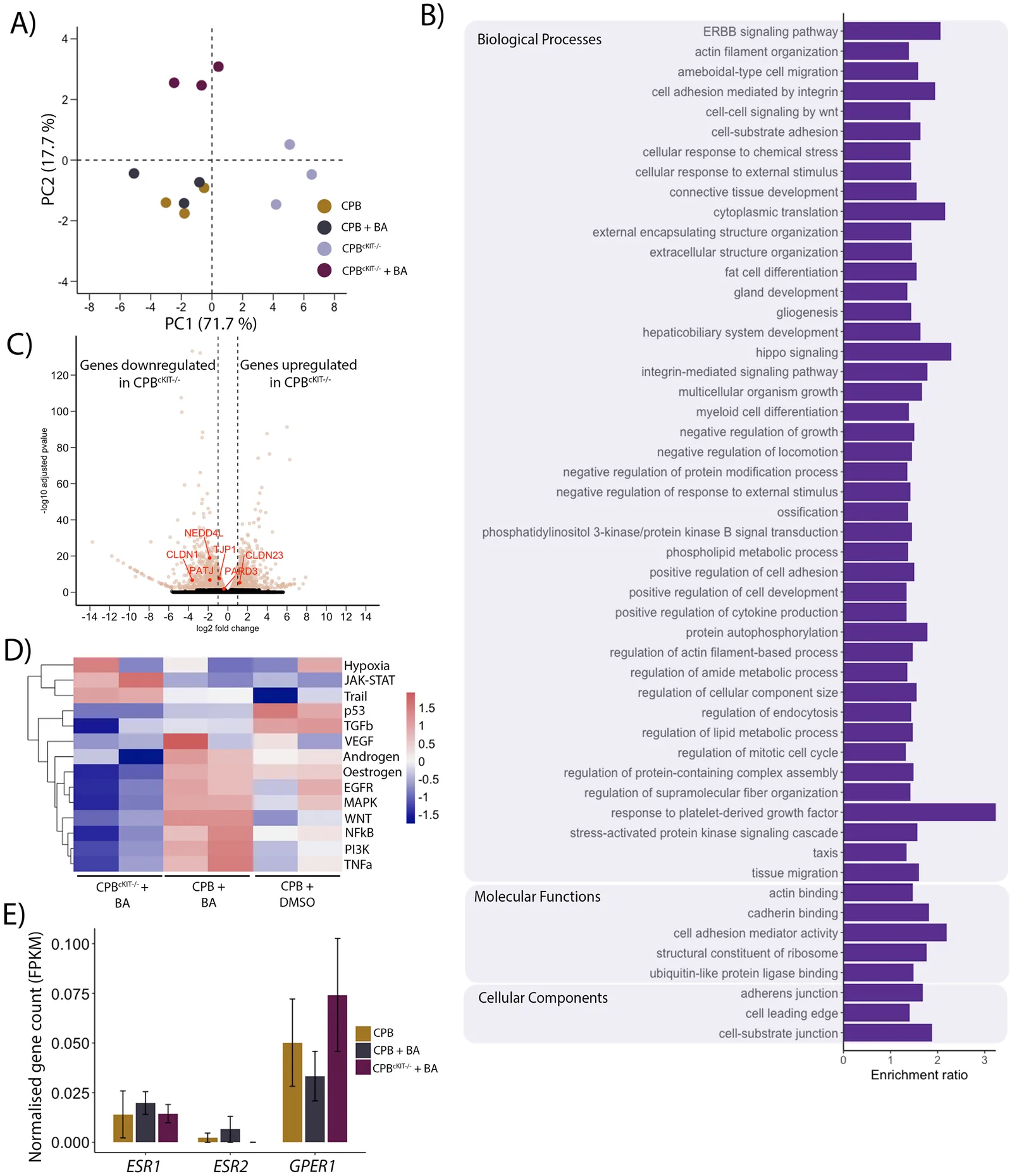
**CPBcKIT-knockoutcells are more sensitive toBAtreatment.** (A) PCA plot of CPB and CPB^cKIT−/−^ cells with and without BA treatment. (B) GO term analysis on DEGs between CPB and CPB^cKIT−/−^ upon BA treatment. Enriched terms (FDR<0.01) are grouped by the GO domain (Biological Processes, Cellular Components, Molecular Functions). (C) Volcano plot comparing the transcriptome of CPB and CPB^cKIT−/−^ upon BA treatment. DEGs (*P*_adj_<0.05) are in pink. TJPs are indicated in red. (D) PROGENy analysis reveals the signalling pathways that were perturbed upon cKIT knockout and BA treatment. Colour key: Pathway activity counts from normalised counts. (E) Expression level of oestrogen receptors in CPB and CPB^cKIT−/−^. *ESR1* gene (ERα), *ESR2* gene (ERβ) and *GPER1* were expressed at low levels.

In patients with GORD, BO tissue is exposed to repeated interaction with acid and bile salts. Periodically fluctuating pH environmental stress conditions require cells to be responsive and robustly protective to ensure tissue integrity and limit access to basal and stromal cells. Recreating patient reflux conditions in cell-based assays is not possible; however, we were interested in observing the cellular response to repeated exposures to acid/bile salt conditions over an extended time period. To assess how exposure to acid and bile salts impacts BO cells, CPB cells were treated with BA (see Materials and Methods for the chemical components of BA) for 3 weeks, three times a week (i.e. a total of nine BA treatments). RNA-seq analysis of CPB and CPB^cKIT−/−^ cells shows that the presence of cKIT impacts the way CPB cells respond to BA insult (Fig. 3). PCA (Fig. 3A) shows that CPB cells with and without BA treatment occupy the same region of the plot, indicating that BA exposure produces only a modest shift in their transcriptional profile. In contrast, CPB^cKIT−/−^ cells with and without BA treatment separate into diagonally opposed quadrants, indicating a larger transcriptional shift in response to the same insult. This difference suggests that deletion of the *KIT* gene not only alters the baseline transcriptional profile but renders the cells transcriptionally more responsive to BA treatment.

GO term analysis (Fig. 3B) of the DEGs in CPB^cKIT−/−^ compared with CPB upon BA treatment shows that these genes are involved in a range of cell functions. However, we noted features common to the previous analysis for the CPB^cKIT−/−^ cells, which included cell migration (tissue migration, taxis, cell-substrate junction, amoeboid-type cell migration, negative regulation of locomotion, cell leading edge), as well as cell adhesion (positive regulation of cell adhesion, cell adhesion mediated by integrin signalling, cell-substrate adhesion, adherens junction) and cell signalling. Of particular interest amongst the DEGs identified in BA-treated CPB and CPB^cKIT−/−^ were the following TJPs: CLDN1, CLDN23, PARD3, PATJ, TJP1 and NEDD4L (Fig. 3C).

### cKIT knockout and oestrogen signalling

Given that cKIT plays a role in initiating signal transduction and that several GO terms are related to signalling pathways, PROGENy analysis was carried out (Schubert et al., 2018). With the top 250 perturbed genes, several pathways were found to distinguish CPB cells from CPB^cKIT−/−^ when they were subjected to BA stress. Hierarchical clustering shows that when CPBs and CPB^cKIT−/−^ are exposed to BA, a number of pathways/functional groups are dramatically affected by the absence of cKIT (Fig. 3D). Several of these have previously been reported as being important in Barrett's cell function, such as mitogen-activated protein kinase (MAPK) and PI3K (e.g. Vona-Davis et al., 2005). Alongside the other receptor-mediated signal transduction, the oestrogen hormone-related signalling pathway (Fuentes and Silveyra, 2019) was observed to be sensitised to BA in the absence of cKIT. In cells where cKIT was deleted, oestrogen signalling was downregulated. The regulation of this hormone is of particular interest because of the established correlation of males and postmenopausal females with predisposition to BO (Dong et al., 2020; Melquist et al., 2022; Beaton et al., 2024).

The three oestrogen receptors ERα, ERβ, and GPER1 (Fuentes and Silveyra, 2019) are expressed in the CPB line. Although the expression levels are low, the transcription levels of the three oestrogen receptors are affected by the presence/absence of cKIT and exposure to BA (Fig. 3E). This is apparently reflected in the gene count for ERα and ERβ, which increases on exposure of CPB to BA; however, in the CPB^cKIT−/−^ cells treated with BA, this decreases.

### Occludin nuclear localisation on BA treatment

How oestrogen signalling contributes to the maintenance of Barrett's condition is not entirely clear. Our RNA-seq data, along with reported data, suggest a possible role in maintaining the integrity of the epithelial tissue layer, which is important in resistance to repeated BA insults. This view is supported by studies that indicate that oestrogen enhances the epithelial barrier function of the oesophagus by potentiating the TJP Occludin, consequently preventing luminal noxious agents from entering the oesophageal epithelium (Masaka et al., 2013; Honda et al., 2016; Qin et al., 2017). Although we do not observe significant differential gene expression for this protein in our RNA-seq data, Occludin forms an intercellular structure with other TJPs including Claudins, PARD3, TJP1 and PATJ, all of which showed differentially regulated expression levels (Fig. 3C).

**Fig. 4.**
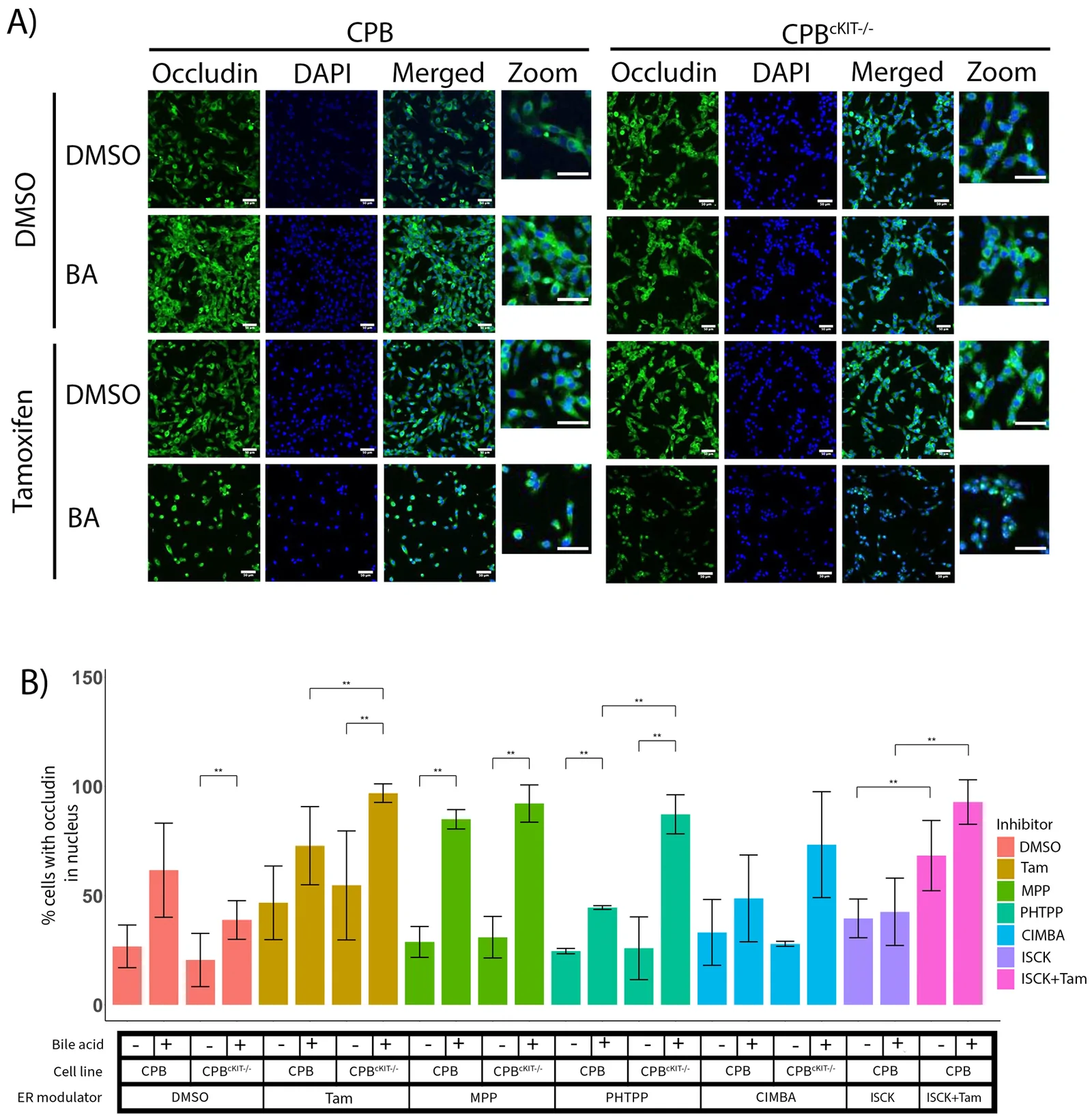
**Occludin is prone to translocate to the nucleus in the absence of cKIT upon disruption oftheoestrogen receptor pathway.** (A) CPB and CPB^cKIT−/−^ cells were immunostained with anti-Occludin to examine Occludin (green) localisation. Nuclei were stained with DAPI (blue). Cells were treated with BA and/or tamoxifen as indicated. Scale bars: 50 μm. (B) CPB and CPB^cKIT−/−^ cells were treated with the oestrogen receptor (ER) modulator tamoxifen (Tam), MPP, PHTPP, CIMBA, or ISCK03 (ISCK), with or without BA; DMSO served as the vehicle control. The percentage of cells with Occludin in the nucleus was counted. **P<0.01. At least three biological replicates were analysed.

To initially examine whether the role of Occludin in oestrogen signalling is preserved in our model and whether it is dependent on cKIT signalling, we investigated the cellular distribution of Occludin. We examined Occludin localisation using immunofluorescence staining (Fig. 4A; Fig. S4). The presence of Occludin was not limited to the plasma membrane where it can perform its primary tight junction formation function. In fact, the broader distribution of Occludin in the cytoplasm and also in the nucleus was striking. In CPB cells, approximately 30% of cells had nuclear distribution of Occludin (Fig. 4B). Stressing of CPB cells through exposure to BA has an impact on Occludin nuclear localisation, increasing it to ∼60%. Exposure to BA appears to be the biggest differentiator between CPB and CPB^cKIT−/−^. In these experiments, oestrogen is available to both CPB and CPB^cKIT−/−^ cells in the culture media. To further explore the impact of oestrogen-related signalling in BA-stressed cells on Occludin localisation, we treated the cells with a general oestrogen receptor inhibitor, tamoxifen (Jordan, 2003). The abrogation of oestrogen receptor signalling in this way led to a marked increase in Occludin nuclear localisation in both cell types, particularly in the CPB^cKIT−/−^ cells, where it was seen in ∼100% of cells. This establishes a link between oestrogen signalling and Occludin localisation in the presence of BA and suggests that, in the absence of cKIT, this effect is amplified. To assess whether the link with Occludin localisation is dependent on a particular receptor, we treated the cells independently with inhibitors which were specific for the individual receptors, i.e. MPP for ERα, PHTPP for ERβ and CIMBA for GPER1 (Sun et al., 2002; DeLeon et al., 2020). The profiles of Occludin nuclear localisation on blocking each receptor are similar, with a consistent increase in Occludin nuclear localisation observed in BA-treated CPB^cKIT−/−^ cells. To confirm that the effect in cKIT knockout was not a clonal effect, the cKIT inhibitor ISCK03 (Na et al., 2007) was added to both CPB cells expressing cKIT. Again, there was an increase in the number of cells with Occludin in the nucleus when cKIT and oestrogen signalling was co-inhibited (Fig. 4B).

## DISCUSSION

BO patients who progress to OAC are predominantly males and postmenopausal females, hence linking its disease progression to reduced oestrogen availability. In this study, we identified the appearance of the RTK cKIT as being important based on its elevated expression in Barrett's tissue but not in normal or OAC tissue. Knocking out *KIT* in an exemplar Barrett's cell line and exposing these cells to BA revealed a link between loss of cKIT downregulating oestrogen signalling and proteins involved in tight junction formation. This can be seen clearly in the nuclear localisation of the TJP Occludin in response to inhibiting oestrogen receptors in cKIT-knockout cells in the presence of BA. In typical BO patients where cKIT prevails, the presence of oestrogen is limited compared to normal female individuals. Any modification of oestrogen signalling through depletion of the RTK under these conditions is likely to have a profound response.

How the loss of cKIT expression in Barrett's tissue can lead to the disruption of tight junction formation is likely to be complex; however, reported literature provides some possible insight. The RTK cKIT is known to signal through multiple downstream pathways known to be linked to oestrogen signalling (including PI3K- and MAPK-mediated pathways; Bosch et al., 2015; Atanaskova et al., 2002; McGlynn et al., 2013). Thus, the loss of cKIT could lead to a concomitant modulation of oestrogen signalling (Rashid et al., 2010; Nie et al., 2018). In this study, this is highlighted by the link between reduced oestrogen signalling and TJPs, specifically Occludin. This protein is known to be affected by oestrogen such that when concentrations are compromised, the loss of Occludin function results in increased permeability in oesophageal epithelium (Masaka et al., 2013; Honda et al., 2016; Qin et al., 2017). Loss of Occludin is linked to adverse features in several tumour types, including oesophageal, colorectal and endometrial adenocarcinomas (Tobioka et al., 2004; Qin et al., 2017; Büyücek et al., 2025).

Our data show that when a BO cell is BA stressed under limiting oestrogen conditions associated with loss of cKIT, the normally plasma-membrane-associated Occludin translocates to the nucleus, compromising tight junction formation. Nuclear localisation of Occludin has been reported in astrocytes (Morgan et al., 2018), indicating a novel function unrelated to tight junction formation. Nuclear expression of Claudin 3, another TJP, has been observed in colorectal cancer (Tokuhara et al., 2018). Occludin can be expressed in several isoforms (Mankertz et al., 2002) and can be cleaved by GPER1 activity (Ulitzky et al., 2016), resulting in independent function; however, the isoform found in the astrocyte nucleus appears to be the same isoform that is found in tight junction formation (Morgan et al., 2018).

In conclusion, the appearance of cKIT in the tissue of BO patients at elevated levels compared to normal or OAC tissue suggests a role for the RTK in limiting cancer promotion or stabilising Barrett's condition. Our data suggest that, under the reduced oestrogen levels typically experienced by BO sufferers (i.e. male and postmenopausal females), the presence of cKIT helps maintain functional oestrogen signalling. As cKIT is diminished, the tight junctions between epithelial cells (symptomatically modified by the condition) required to provide a robust barrier to acid and bile salt exposure are compromised by the movement of Occludin to the cytoplasm and nucleus. The potential for cKIT to be a viable marker for Barrett's progression to OAC and whether therapeutic intervention can be effected in late-stage HGD tissue via cKIT supplementation are yet to be investigated.

## MATERIALS AND METHODS

### Cell culture

CPB, CPC and CPD cells were grown in complete media (Fisher Scientific, 12539079) supplemented with 5% FBS. Cells were split or fed every 3 days.

CPB cells were used for the bulk of the experimental studies. These were obtained from ATCC^®^ and alternatively known as CP-52731, the immortalised, adherent, hypodiploid CPB cell line derived in 1994 from a region of HGD present in a male donor. As with the CPA cell line, CPB cells were immortalised via transduction with the pLXN-hTERT expression vector. Numerous derivative chromosomes are present but differ by passage number, with the tetraploid population increasing from approximately 18% up to 50% at higher passages. These include the derivative chromosomes der(1)t(1;17)(q42;q21), add(8)(p11.2), der(9)t(9;14)(q10;q10), add(12)(q13), add(15)(q24.3), add(17)(p11.2), del(19)(p13.1) and del(21)(q22.1). All cultures were confirmed negative for mycoplasma contamination at least once every 2 months using the PCR-based LookOut^®^ Mycoplasma Detection Kit (MP0035).

### Drug and BA treatment

Experiments were carried out in triplicate. The complete medium was adjusted to pH 4.0 using hydrochloric acid. Bile salts were dissolved at 100 mM in DMSO and used at a final concentration of 100 μM. The following bile salts were used: glycocholic acid (Sigma, 360512), taurocholic acid (Sigma, T4009), glycodeoxycholic acid (Insight Biotechnology, sc-280755), glycochenodeoxycholic acid (Sigma, 50534), and deoxycholic acid (Sigma, D2510). The final BA medium was filtered and sterilised using a 0.22 μm filter before use. The control medium was prepared similarly without acidification, and DMSO was used instead of bile salts.

Drugs were added to cells overnight. The medium was refreshed on the day of the experiment, and cells were treated with drugs again 2 h prior to the start of the experiment. 10 mM of drugs dissolved in DMSO was used: tamoxifen (Merck, T5648-1G), ISCK03 (Stratech Scientific Ltd, S2070-SEL-25 mg), MPP dihydrochloride hydrate (Merck, M7068-10MG), PHTPP (Cambridge Bioscience, HY-103456-10 mg), and CIMBA hydrochloride (Fisher Scientific, 18717706). Cells were treated with acidified complete media supplemented with bile salts for 10 min before they were washed with PBS. Cells were allowed to recover in complete media at normal pH for 60 min before sample collection.

### cKIT CRISPR knockout

Briefly, oligos containing the sequences matching exon 3 were cloned into the All-in-One-GFP (Addgene plasmid #74119). CPB cells were transfected with the All-in-One-GFP targeting *KIT* specifically, alongside a repair template, Cttttaaaaagtgttttcagtgtctgtgaccagccattccaactactgattttggatatgcttctatagatcctgtaaatgatagatagttgttgaccgctccttgtatgggaaagaagacaacgacacgctagtccgctgtcctctcacagacccagaagtga, using Lipofectamine 3000. A range of DNA:Lipofectamine 3000 ratios was used. The one with the highest transfection efficiency was carried forward for downstream screening. Single clones were isolated by serial dilution into 96-well plates. To identify clones in which the *KIT* gene was successfully edited, the 96-well plates were copied. When the cells were confluent, the cells were lysed in 20 μl of lysis buffer [50 mM KCl, 10 mM Tris-HCl (pH 8.3), 2.5 mM MgCl_2_, 0.45% NP-40, 0.45% Tween-20] supplemented with 0.4 mg/ml of proteinase K. To prepare the DNA for PCR analysis, lysate was heated at 65°C for 30 min, followed by 95°C for 20 min. 2 μl of lysate was used in the subsequent 20 μl PCR. Clones with indels were expanded and sequenced to identify the alteration. The clone designated P2B3 was used in this work. It contains complex indels spanning the 3′ end of exon 3 (ACCAGCCATTCCAACTACTGATTTTGTGACTTGATTGCAAACATCACGAAGGTGTTTCTGAGAATGCTTCTGTCTAGATTTTCTTTGAAGACATTCCCGTTTCCAACGAAATCCTCACAGCTATCCAAATATCCTCTTGCAGATTCTACAAAAAGTGTGGTTCAAAACTGCTGTATCAAAAGAATGGATCAACACTGTTAGTTGAGTACCCACATCACAAACGTGATTCTCAGAATGCTTCTGTCTAGAAGACAACGACACGCTGGTCCGCTGTCCTCTCACA) that results in a STOP codon.

### mRNA sequencing and data analysis

RNA was extracted using the Direct-zol RNA miniprep kit (Zymo, R2050). RNA library preparation and sequencing were carried out by Novogene. PolyA enrichment was used to enrich mRNA. With the NovoMagic interface, DESeq2 was used for normalisation and to identify DEGs. Data were visualised using RStudio. GO term analysis was performed using https://www.webgestalt.org/. Over-representation analysis was used as the enrichment method, with *Homo sapiens* as the selected organism and the GO Biological Process/Molecular Function/Cellular Component categories as the functional database. Gene lists were submitted as gene symbols and assessed against the genome protein-coding reference set. For each analysis, functional categories were required to contain between five and 2000 genes, and enrichment significance was determined using the Benjamini-Hochberg false discovery rate (FDR), with a threshold of <0.05.

### Immunostaining

Cells were seeded at 100,000 cells/well in a 24-well plate containing glass coverslips on day 1. On day 4, inhibitors were applied overnight. On day 5 (the day of the experiment), inhibitors were freshly applied for 60 min before cells were treated with BA for 10 min. Cells were then washed with PBS once before a 60-min recovery period with complete media. Inhibitors/DMSO were present throughout the experiment except during the PBS wash. After the recovery period, cells were washed once with PBS and fixed with 4% PFA for 20 min at room temperature. Samples were then washed two to three times with PBS. This was followed by permeabilisation and blocking in PBST (PBS with 0.1% Triton X-100) supplemented with 3% BSA either overnight at 4°C or for 30 min at room temperature. Samples were incubated in primary antibodies diluted in PBST with 3% BSA overnight at 4°C or 2 h at room temperature. Excess antibodies were removed by washing three times for 10 min in PBST. Samples were then incubated in fluorescent dye-conjugated secondary antibody and 1 mg/ml of DAPI diluted in PBST for 1 h at room temperature, protected from light. Samples were washed three times in PBS +0.1% Triton X-100 for 5 min each, protected from light, before being mounted on slides using a mounting solution (VECTASHIELD, H-1000). Images were taken on Leica confocal microscopes.

### Western blotting

Cells were lysed in lysis buffer [50 mM HEPES, 50 mM NaCl, 1 mM EGTA, 10% (w/v) glycerol, 1 mM sodium orthovanadate, 10 mM sodium fluoride, 0.1% NP-40, supplemented with protease inhibitors] and cleared of cell debris via centrifugation. Samples were then analysed by western blotting. Antibodies used were anti-cKIT (Proteintech, 18696-1) and tubulin (11224-1).

### Crystal Violet assay

20,000 cells/well were seeded in a six-well plate the day before the assay. On the day of assay, cells were washed with PBS before being fixed with 4% PFA for 20 min at room temperature. Cells were then washed with PBS three times, followed by one brief wash with water. Cells were stained in 0.2% Crystal Violet (dissolved in 20% methanol) at room temperature for 30 min. Stained cells were washed with PBS three times. Cells were left in 1 ml PBS and imaged on an EVOS microscope immediately.

## Supplementary Material



10.1242/biolopen.062802_sup1 Supplementary information
