# Supplementary material for: Regulation of oestrogen signalling by the receptor tyrosine kinase cKIT stabilises Barrett's oesophagus pre-malignant state
Source: Biol Open. 2026 Sep 8;15(9):bio062802. doi: 10.1242/bio.062802 (PMC13602374; doi:10.1242/bio.062802)
Supplement: Supplementary file 1 [file biolopen-15-062802-s1.pdf]

Supplemental figure 1

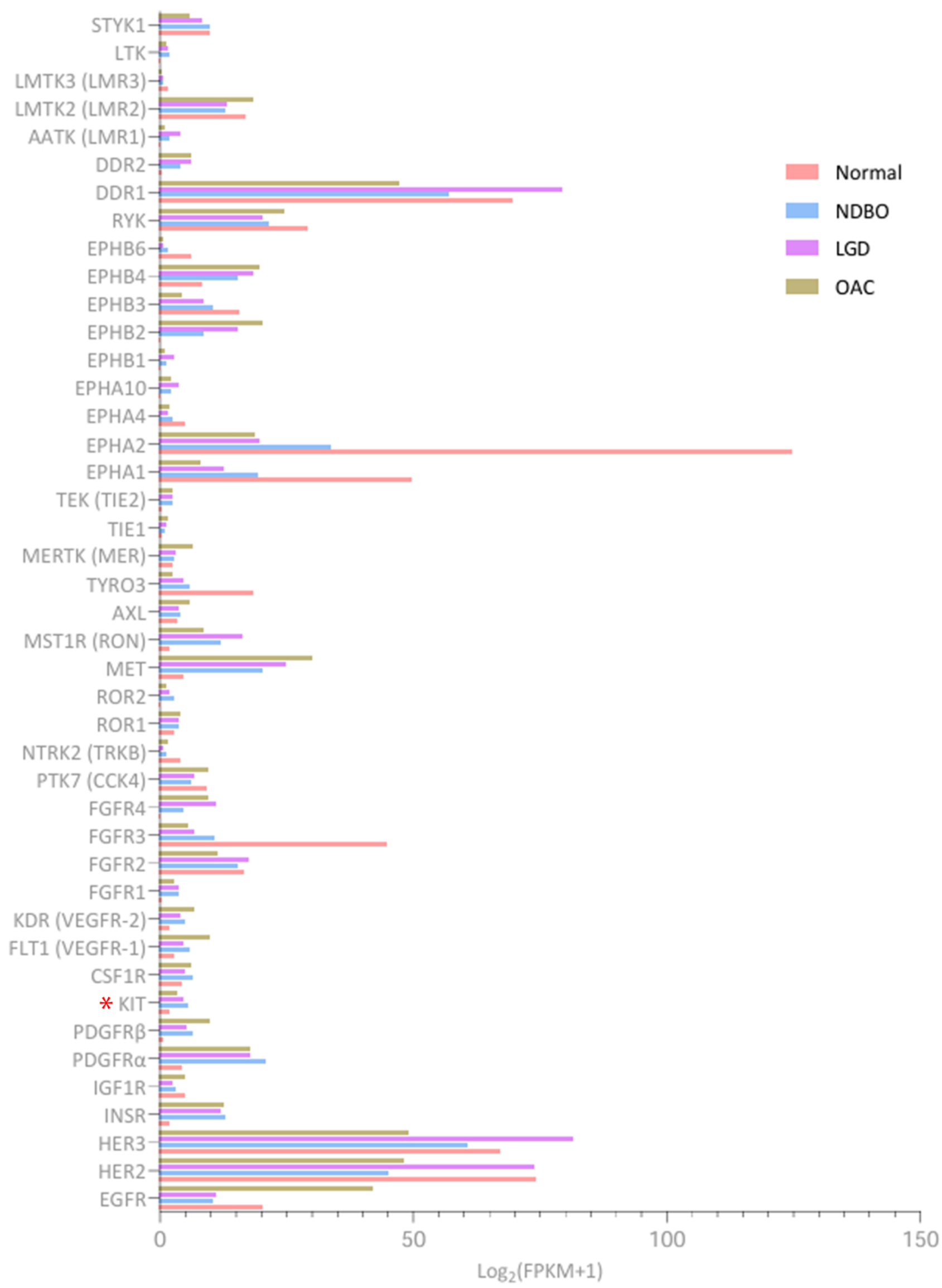

Fig. S1. mRNA expression of RTKs in normal, non-dysplastic Barrett's oesophagus (NDBO), low-grade dysplasia (LGD) and oesophageal adenocarcinoma (OAC) tissues in a patient dataset derived from Maag et al. (2017). RTKs for which FPKM values were less than 1 across all tissue types are unlikely to be expressed and were removed from the graph. Red asterisk marks cKIT (labelled as KIT).

Biology Open • Supplementary information

Supplemental Figure 2

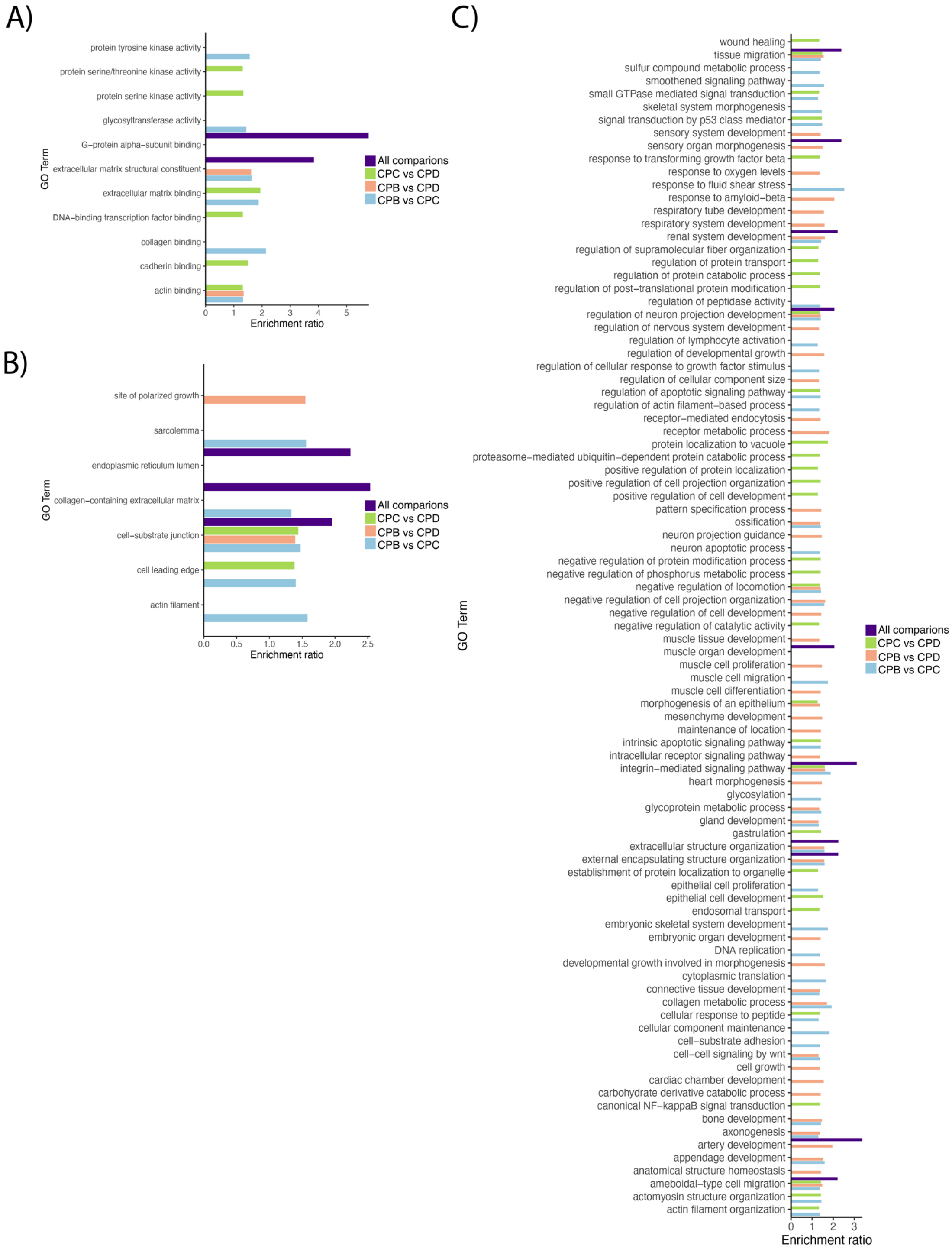

Fig. S2. GO term analysis of CPB, CPC and CPD.

GO term analysis (FDR < 0.01) of DEGs (padj < 0.05) in pairwise comparisons of CPB vs CPC, CPB vs CPD, CPC vs CPD and among all cell lines. a) Molecular function, b) cellular component and c) biologicial process.

Biology Open • Supplementary information

Supplemental figure 3

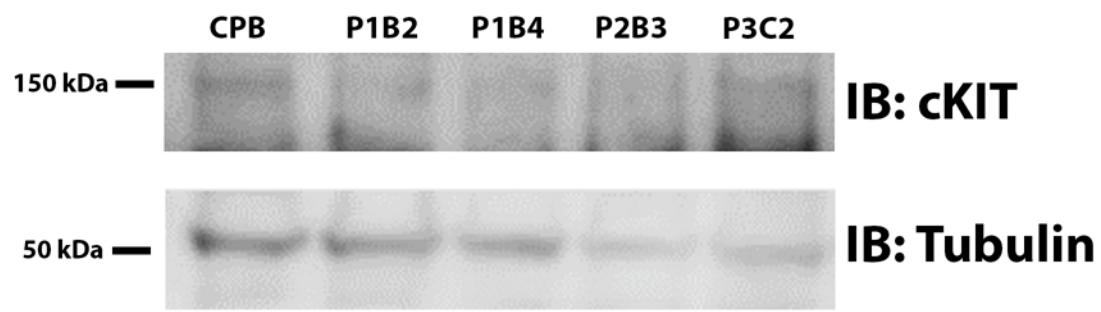

Fig. S3. Expression level of cKIT in CPB CRISPR-edited cell lines.
cKIT protein expression level was examined for several CRISPR clones. P2B3 showed clear absence of cKIT expression and was therefore adopted for experimentation.

Supplemental figure 4

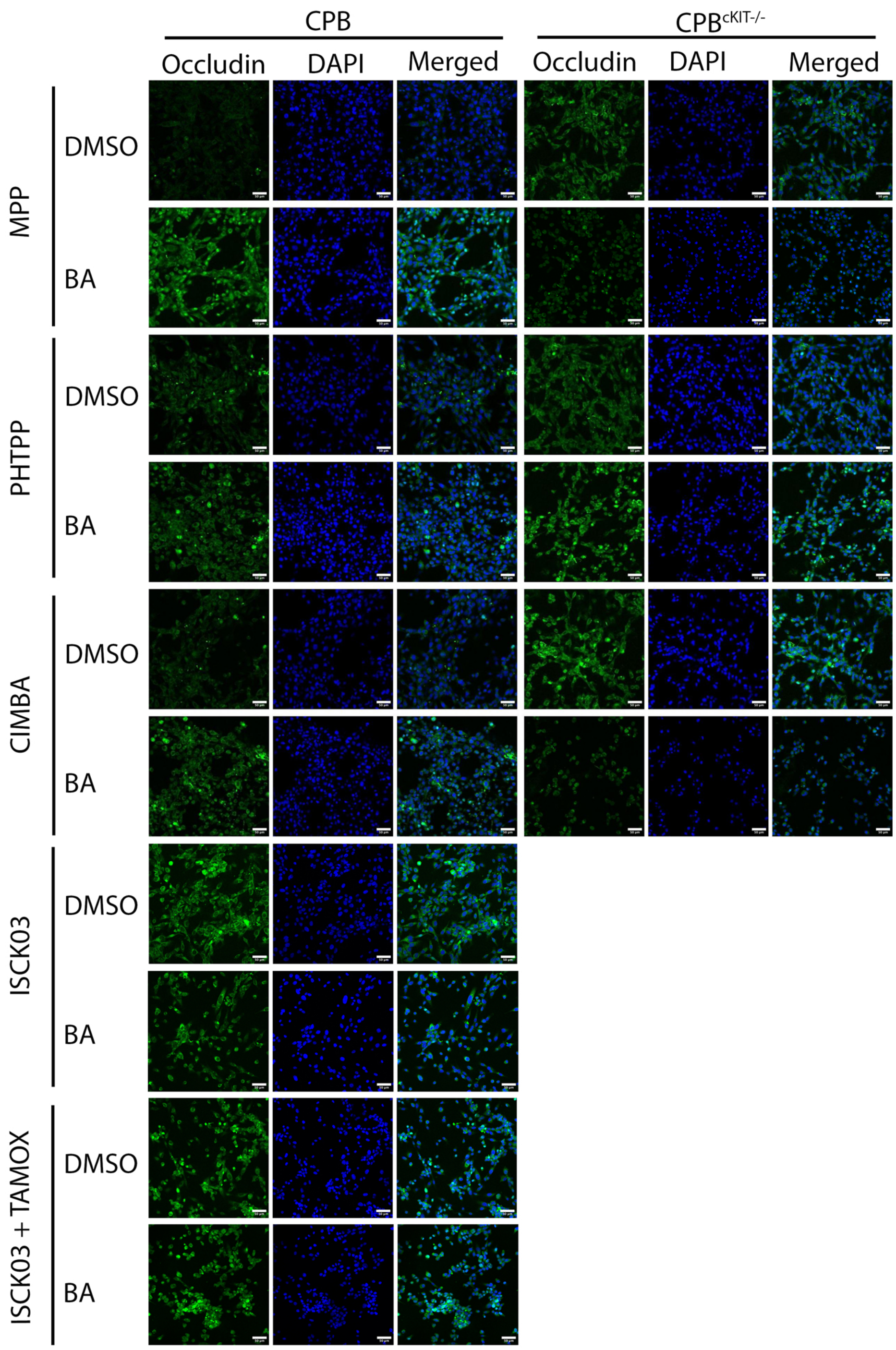

Fig. S4. Occludin is more prone to translocate to the nucleus in the absence of cKIT upon disruption of oestrogen receptor pathway.

CPB and CPB$^{cKIT-/-}$ cells were immunostained with anti-Occludin to examine occludin (green) localisation. Nuclei were stained by DAPI (blue). Cells were treated with bile acid and/or MPP, PHTPP, or CIMBA as indicated. cKIT inhibitor ISCK was also used. Scale bar = 50 µm.

Biology Open • Supplementary information
