# Supplementary material for: Regulation of oestrogen signalling by the receptor tyrosine kinase cKIT stabilises Barrett's oesophagus pre-malignant state
Source: Biol Open. 2026 Sep 8;15(9):bio062802. doi: 10.1242/bio.062802 (PMC13602374; doi:10.1242/bio.062802)
Supplement: Supplementary file 2 [file biolopen-15-062802_review_history.pdf]

Regulation of oestrogen signalling by the receptor tyrosine kinase cKIT stabilises the Barrett's Oesophagus pre-malignant state

Kin Man Suen, Safoura Zahed Mohajerani, Poppy G. Tyrer, Henry W. Clark, Christopher M. Jones and John. E. Ladbury

10.1242/bio.062802

Editor: Catherine L. Jackson

Review timeline

Original submission:	7 July 2026
Editorial decision:	15 July 2026
First revision received:	28 July 2026
Accepted:	3 August 2026

Original submission

First decision letter

MS ID#: bio.062802

MS Title: Regulation of oestrogen signalling by the receptor tyrosine kinase cKIT stabilises the Barrett's Oesophagus pre-malignant state

Authors: Kin Man Suen, Safoura Zahed Mohajerani, Poppy G. Tyrer, Henry W. Clark, Christopher M. Jones and John. E. Ladbury

I have now reached a decision on the above manuscript.

The reviewer reports are shown at the bottom of this email.

As you will see, the reviewers raised a number of substantial criticisms that prevent me from accepting the paper at this stage.

They suggest, however, that a revised version might prove acceptable, if you can address their concerns. If you think that you can deal satisfactorily with the criticisms on revision, I would be pleased to see a revised manuscript. We would then return it to the reviewers.

At this stage, we also ask you to ensure your manuscript complies with our formatting guidelines. Provided you are able to fully address the referees' comments, we are positive about publication of your paper (we accept over 95% of revision submissions) and therefore hope you won't mind any extra work involved in reformatting your manuscript at this point.

Please upload both a 'clean' version of your Word file, along with a highlighted version clearly showing where you have made changes in the revised manuscript. Please avoid using 'Track changes' in Word files as these are lost in PDF conversion.

I should be grateful if you would also provide a point-by-point response detailing how you have dealt with the points raised by the reviewers in the 'Response to Reviewers' box. Please attend to all of the reviewers' comments. If you do not agree with any of their criticisms or suggestions please explain clearly why this is so.

© 2026. Published by The Company of Biologists under the terms of the Creative Commons Attribution License (https://creativecommons.org/licenses/by/4.0/).

Reviewer 1

Comments for the author

1. describe your procedures more for performing the cell culture, how many plates did you use? How many times did you do the experiment? How did you do the statistical analysis? What is the GO analysis (ie how was it done?) raw data?
2. abstract: although this is clarified later in the introduction, there needs to be clarity about "stable to cancer promotion" - it could be interpreted as either cancer promotion or cancer inhibiting based on the way it is currently phrased
3. intro: article discusses driver mutations and changes to gene transcription and protein expression. I was to clarify that you are not referring to detecting passenger mutations in this analysis correct? If driver mutations are going to be discussed, it would make sense to also mention passenger mutations.
4. intro: page 5 line 18 - do you mean progression to OAC?
5. results: line 48 "howevers?" - this whole sentence needs to be reworked, it is difficult to understand
6. results: page 7 - what is GORD? please specify this acronym
7. page 8: comma between BA, a number of ....
8. general note: commas need to be inserted into a lot of places, it will help with the clarity
9. page 8: a lot of time is spent mentioning that estrogen and males/post meno females. I would recommend making a sharper connection with this at some point in the article. Why is this important for our study? What do we know about normalling signalling that makes this finding so significant
10. Figure 1C, 2C, and 3B - plots need to be clarified. they are not well labeled and they are comparing different cell lines but no indication of how this is being done in the figure captions or on the graph itself.

Reviewer 2

Comments for the author

The authors claim to demonstrate the role of cKIT in the maintenance of Barrett's oesophagus (BO), proposing that cKIT contributes to stabilisation of the Barrett's phenotype through modulation of oestrogen signalling and maintenance of epithelial tight junction integrity. Although the study aims to address an important question in Barrett's oesophagus biology, the manuscript, in its current form, is highly speculative and frequently overstates the conclusions that can be drawn from the data. While the data were obtained using complementary molecular approaches, they do not directly demonstrate a role for cKIT in the maintenance of BO or in delaying or preventing progression to oesophageal adenocarcinoma (OAC).

Specific Comments

1. The conclusions remain highly speculative and extend beyond the evidence presented. Although the data support changes in gene expression, pathway activity and protein localisation following KIT knock-out, they do not directly demonstrate that cKIT maintains the Barrett's phenotype or delays/prevents progression to oesophageal adenocarcinoma. Data demonstrating increased transformation. dysplasia and tumour formation are all missing to support this conclusion. These experiments are required to justify the conclusion and even the title. As its stand its misleading
2. The authors state: "Based on previously reported meta-analysis of patient data (Maag et al., 2017, Jones, 2021)we were able to interrogate the role of all known RTKs in BO by comparison with data from normal and OAC tissue. Amongst all the RTKs reported, cKIT showed low overall expression,howevers unlike the other RTKs, showed elevated expression only in HGD Barrett's tissue" It is unclear which data are the author drawing this conclusion from. Clarification is required
3. The evidence supporting the KIT knock out (line 58) at this point is not sufficiently convincing. The authors should repeat the western blot experiment and also provide the complete, uncropped blots as supplementary data to allow assessment of band specificity and data quality.
4. The authors' state: "This suggests that the deletion of the KIT gene results in a different transcriptional profile which makes cells sensitive to BA treatment." This conclusion is not supported by the data presented. The PCA presented only demonstrates differences in

© 2026. Published by The Company of Biologists under the terms of the Creative Commons Attribution License (https://creativecommons.org/licenses/by/4.0/).

transcriptional responses following BA treatment but does not provide any evidence or suggestion that KIT-KO cells are more sensitive to bile acid exposure. To make such claim appropriate functional assays such as cell viability, proliferation, apoptosis assay is required.
5. With Figure 3E, although differences in gene expression are somewhat apparent, but it lacks statistical analysis. Without appropriate statistical testing, it is difficult to determine whether the observed differences are biologically meaningful.
6. The Occludin localisation in Figure 4 which appears to be major claim of this paper requires further supportive experiments. Although the immunofluorescence images are consistent with the authors' description. The images are of relatively low magnification making accurate assessment of subcellular localisation difficult. I recommend that the authors validate these findings using subcellular fractionation followed by Western blot analysis for Occludin, together with appropriate nuclear and cytoplasmic marker proteins. Such experiments would provide considerably stronger evidence for the proposed nuclear translocation of Occludin.
7. I will also recommend that, to strongly validate the role of cKIT, authors should re-express KIT in KIT-KO cells and demonstrate if this restore the lost functions or changes in gene expression observed in KIT Ko cells

<u>Reviewer 3</u>

Comments for the author

In this manuscript, the authors investigate the mechanism in the pathogenesis of Barrett's Oesophagus, demonstrating how the receptor tyrosine kinase cKIT cross-talks with and regulates estrogen receptor signaling to stabilize a pre-malignant tissue state. By combining transcriptomic profiling, bioinformatic pathway modeling, and targeted pharmacological assays using bile acid treatments and selective estrogen receptor modulators, the study addresses a highly interesting and clinically relevant hypothesis. The overall experimental design is logically structured and the bioinformatic road mapping is sound; however, the technical execution and data presentation currently fall somewhat short of the standards required to support the authors' central claims. Significant revisions are required to resolve these critical technical flaws before the manuscript's conclusions can be fully verified.

Major Revisions

1. Technical Failure of Knockout Validation and Missing Quantification (Supplemental Figure 3)

* Issue: The Western blot intended to verify the CRISPR-mediated knockout of cKIT in single-cell clones (Supplemental Figure 3) suffers from a fatal biochemical validation flaw. The housekeeping loading control (IB: Tubulin) is profoundly uneven across the gel; it shows a strong band for parental CPB, P1B2, and P1B4, but is severely depleted and nearly invisible in the exact lane for the selected knockout clone (P2B3) and clone P3C2. Furthermore, no densitometric quantification is provided. The blot also appears to be very closely cut.

* Comment to Author: A uniform loading control is mandatory to conclude that a target band's absence is caused by a genetic knockout rather than an underloaded lane or sample degradation. Because the Tubulin signal drops off precipitously in the P2B3 lane, the apparent absence of the 150 kDa cKIT band cannot be trusted. Since this single clone forms the absolute foundation for every phenotypic essay in Figures 2, 3, and 4, the core conclusions are currently unverified. The authors must repeat this Western blot ensuring equalized total protein loading with robust, uniform Tubulin bands across all lanes, alongside relative densitometric quantification with replicates, or provide definitive Sanger sequencing/ICE genomic confirmation of the out-of-frame knockout.

2. Lack of Statistical Significance and Massive Variance in Target Expression (Figure 3E)
* Issue: The manuscript claims that cKIT knockout explicitly modulates Estrogen Receptor signaling. While the PROGENY pathway inference (Figure 3D) hints at this trend, the actual, direct gene expression levels (FPKM values) shown in Figure 3E tell a contradictory story. The error bars for ESR1 and GPER1 are highly expansive, heavily overlapping between the control CPB, CPB + BA, and CPB cKIT-/- + BA conditions. Shockingly, there are no statistical significance markings (p-values or asterisks) anywhere on this chart.

© 2026. Published by The Company of Biologists under the terms of the Creative Commons Attribution License (https://creativecommons.org/licenses/by/4.0/).

* Comment to Author: A claim of specific pathway regulation cannot be substantiated when the individual gene expression levels of those target receptors lack statistical significance and exhibit overlapping variances. Please perform an appropriate statistical test (e.g., ANOVA with post-hoc corrections) on the data in Figure 3E and clearly display the resulting significance markers. If the difference is non-significant due to variance, the overarching claim regarding direct estrogen receptor regulation must be heavily scaled back.

3. Morphological Representation Concerns (Figure 2A)
* Issue/Comment to Author: While the box plots on the right of Figure 2A demonstrate a statistically significant shift in cell area and circularity, the microscopy panel on the left is composed entirely of heavily cropped individual single cells. Presenting singular isolated cells creates an opening for confirmation bias. Please replace these individual crops with wide-field, representative phase-contrast views of the cell monolayer cultures to accurately communicate the uniform phenotypic shifts of the population. Alternatively, the singular isolated crops could be used as zoomed-in blocks placed above the wide-field images.

4. Mismatch in Figure 3 Text, Panel Layout, and Legend
* Issue/Comment to Author: There is a major layout breakdown in Figure 3. The figure features panels A through E. However, the Figure 3 legend skips panel C (the volcano plot) completely in its descriptive lettering sequence and conflates the subsequent panels, incorrectly labeling the PROGENY heatmap as (c) and the Estrogen Receptor expression chart as (d). Please carefully re-align the Figure 3 legend text to perfectly mirror panels A, B, C, D, and E.

Minor Revisions

1. Inconsistent Nomenclature and Labeling Conflicts (Figure 4B)
* Issue/Comment to Author: Figure 4B contains conflicting text elements that hamper scannability. The color-coded legend box on the right categorizes the independent variables as an "Inhibitor", while the data matrix underneath the x-axis categorizes them as an "ER modulator". Additionally, the compound names are inconsistently abbreviated between the two spaces (e.g., TAMOX vs Tamoxifen; ISCK vs ISCK03; TAMOX+ISCK vs ISC+Tam). Please choose a single uniform naming convention and apply it to both the legend box and the x-axis data table.

2. Textual and Graphical Errors (Figures 1, 2, and 3)
* Issue/Comment to Author: Please fix the following errors during manuscript revision:
o Figure 1B: The bottom circle of the Venn diagram contains a typo, reading "CPC cv CPD" instead of "CPC vs CPD".
o Figure 2A Legend: The text inside the legend for Figure 2a completely swaps the directional layout of the panel. The cell images are on the left (not the right), and the quantitative boxplots are on the right (not the left). Additionally, the sentence ends abruptly with an unedited fragment ("...staining of."), and falsely claims the cells are stained with crystal violet when they appear to be standard gray phase-contrast images. Please rewrite this legend for accuracy, clarity, and grammatical completeness.
o Results Text: Clean up typos such as "amongst the DEGsS which for BA treated..." to "amongst the DEGs which were treated..."

Reviewer's Responses to Questions

Experimental quality
Are experiments performed using appropriate methods that will answer the question (or test the hypothesis or support the observations) posed by the authors? Is the right tool used for the job?
If 'No', please indicate reasons in Comments for Author box below.
Reviewer #1:
- Yes

Reviewer #2:
- Yes

Reviewer #3:

© 2026. Published by The Company of Biologists under the terms of the Creative Commons Attribution License (https://creativecommons.org/licenses/by/4.0/).

- No

Were the data analyzed using appropriate statistical tests?
If 'No', please indicate reasons in Comments for Author box below.
Reviewer #1:
- Yes

Reviewer #2:
- No

Reviewer #3:
- No

Reproducibility
Were experiments performed using adequate number of biological replicates?
If 'No', please indicate reasons in Comments for Author box below.
Reviewer #1:
- No

Reviewer #2:
- Yes

Reviewer #3:
- Yes

Is there sufficient raw data to assess the rigor of the analysis?
If 'No', please indicate reasons in Comments for Author box below.
Reviewer #1:
- No

Reviewer #2:
- No

Reviewer #3:
- No

Does the methods section provide sufficient detail to permit reproducibility?
If 'No', please indicate reasons in Comments for Author box below.
Reviewer #1:
- No

Reviewer #2:
- Yes

Reviewer #3:
- Yes

Completeness
Are the manuscript's conclusions supported by the data?
If 'No', please indicate reasons in Comments for Author box below.
Reviewer #1:
- Yes

Reviewer #2:
- No

Reviewer #3:
- No

© 2026. Published by The Company of Biologists under the terms of the Creative Commons Attribution License (https://creativecommons.org/licenses/by/4.0/).

Scholarship
Do the authors cite and discuss the merits of data that would argue for and against their conclusion?
If 'No', please indicate reasons in Comments for Author box below.
Reviewer #1:
- Yes

Reviewer #2:
- Yes

Reviewer #3:
- Yes

Does the manuscript title & abstract accurately reflect the contents of the manuscript, without hyperbole?
If 'No', please indicate reasons in Comments for Author box below.
Reviewer #1:
- Yes

Reviewer #2:
- No

Reviewer #3:
- Yes

First revision

Author response to reviewers' comments

We are very grateful to the Reviewers for their detailed reading of the manuscript and the provision of helpful and constructive comments. Each comment has been carefully read and our responses to these are detailed below:

Comments from the Reviewers:

Reviewer 1:

1. describe your procedures more for performing the cell culture, how many plates did you use? How many times did you do the experiment? How did you do the statistical analysis? What is the GO analysis (ie how was it done?) raw data?

We thank the reviewer for raising this important point regarding experimental conditions etc. We have updated the cell culture conditions as described in the Materials and Methods under the 'Cell culture' and 'Drug and bile acid treatment' paragraphs. The number of plates used varied according to the amount of RNA required by the sequencing. Experiments were carried out in triplicate, as indicated in the GEO submission (GEO accession number is GSE339303). Read mapping was carried out using Novogene's Novomagic analysis platform, and DESeq2 was used within Novomagic for normalisation and identification of differentially expressed genes. Subsequent analyses were performed by the authors using the DEG lists generated by this pipeline. We have now added a description of the GO term analysis parameters to the Materials and Methods in the 'mRNA sequencing and data analysis' paragraph. Raw data have been deposited in GEO, as stated in the 'Data Availability' section.

2. abstract: although this is clarified later in the introduction, there needs to be clarity about "stable to cancer promotion" - it could be interpreted as either cancer promotion or cancer inhibiting based on the way it is currently phrased

© 2026. Published by The Company of Biologists under the terms of the Creative Commons Attribution License (https://creativecommons.org/licenses/by/4.0/).

In this work we wanted to frame a different way of thinking about cancer development than is usual. In most studies of cancer the pre-malignant state is investigated to establish the factors (promoters) that drive cells to become cancerous. Here we attempted to identify the factors that are required to sustain the pre-cancerous state which prevail in the majority of Barrett's sufferers. By identifying these we might be able to indicate ways which could prevent Barrett's patients developing OAC and provide markers for the different levels of progression of BO. We have added to the Abstract to clarify that only a low percentage of Barrett's patients develop oesophageal adenocarcinoma. Therefore, the requirements for cancer promotion are not usually met.

3. intro: article discusses driver mutations and changes to gene transcription and protein expression. I was to clarify that you are not referring to detecting passenger mutations in this analysis correct? If driver mutations are going to be discussed, it would make sense to also mention passenger mutations.

This is a good point raised by the Reviewer. We state in the Introduction "Despite BO and OAC being associated with high mutational burden (see TCGA data: https://www.cancer.gov/ccg/research/genome-sequencing/tcga/using-tcga-data/citing), the number of recurrent point mutations in cells is low, inferring that the impact of driver mutations is outweighed by changes in gene transcription levels and protein expression." This highlights that driver mutations are not the primary route to cells becoming cancerous, but rather that changes in transcriptional levels, and hence protein concentrations, are key.

4. intro: page 5 line 18 - do you mean progression to OAC?

Here we mean promotion. Typically cancer is described in four states - initiation, promotion, progression and resistance. We view the Barrett's condition as initiated by some key mutations, but promotion factors are required to produce the normal cancer hallmarks.

5. results: line 48 "howevers?" - this whole sentence needs to be reworked, it is difficult to understand

We thank the Reviewer for pointing this out. We had failed to include reference to Supplementary Fig 1 which clarifies the message of this sentence. This sentence has been modified.

6. results: page 7 - what is GORD? please specify this acronym

The acronym GORD is now highlighted in the Introduction. It stands for gastr**o**esophageal **r**eflux **d**isease.

7. page 8: comma between BA, a number of ....

Corrected.

8. general note: commas need to be inserted into a lot of places, it will help with the clarity

We have reread the manuscript and added commas where appropriate.

9. page 8: a lot of time is spent mentioning that estrogen and males/post meno females. I would recommend making a sharper connection with this at some point in the article. Why is this important for our study? What do we know about normalling signalling that makes this finding so significant

This is an important point. We highlight this issue in the Introduction: "Knocking out the *KIT* gene had a significant impact on the expression genes associated with oestrogen signalling in the presence of BA, thus providing a link to BO via the known prevalence in male and post-menopausal female patients." We also add further clarification in the Results: "In cells where cKIT was deleted oestrogen signalling was downregulated. The regulation of this hormone is of particular interest because of the established correlation of male and post-menopausal females with predisposition to BO (Dong et al., 2020; Melquist et al., 2022; Beaton et al., 2024)." Finally, we add further

© 2026. Published by The Company of Biologists under the terms of the Creative Commons Attribution License (https://creativecommons.org/licenses/by/4.0/).

sharpness to the issue in the Discussion: "BO patients who progress to OAC are predominantly males and post-menopausal females, hence linking its disease progression to reduced oestrogen availability." and " Our data suggest that, under the reduced oestrogen levels typically experienced by BO sufferers (i.e. male and post-menopausal females), the presence of cKIT helps to maintain functional oestrogen signalling." Hopefully, these numerous mentions of the link should suffice to address the Reviewer's comment.

10. Figure 1C, 2C, and 3B - plots need to be clarified. they are not well labeled and they are comparing different cell lines but no indication of how this is being done in the figure captions or on the graph itself.

We thank the reviewer for pointing out the insufficiency in our figure legends. We have added the relevant details to the legends for Figures 1C, 2C and 3B. For example, the legend for Figure 1C now states that the analysis was carried out on the 918 differentially expressed genes shared across all three pairwise comparisons between the CPB, CPC and CPD cell lines.

Reviewer 2:

The authors claim to demonstrate the role of cKIT in the maintenance of Barrett's oesophagus (BO), proposing that cKIT contributes to stabilisation of the Barrett's phenotype through modulation of oestrogen signalling and maintenance of epithelial tight junction integrity. Although the study aims to address an important question in Barrett's oesophagus biology, the manuscript, in its current form, is highly speculative and frequently overstates the conclusions that can be drawn from the data. While the data were obtained using complementary molecular approaches, they do not directly demonstrate a role for cKIT in the maintenance of BO or in delaying or preventing progression to oesophageal adenocarcinoma (OAC).

We would like to thank the Reviewer for their comments and we understand the points that have been made. As the Reviewer notes, the manuscript brings together data from complementary molecular approaches to investigate the connection between the receptor tyrosine kinase, cKIT, and a potential role in Barrett's Oesophagus. Our approach has been different to many studies in this area in that, rather than highlighting the drivers of the BO to OAC promotion, we have tried to determine the molecular components that could be responsible for maintaining the Barrett's condition. Clearly, this approach is not designed to find possible targets that might lead to curing OAC, but rather understanding features that sustain the pre-malignant state. Since the vast majority of BO patients do not progress to OAC, these features may be important as markers for progression, but also point to ways in which BO can be managed to prevent OAC. Since, this approach is novel, it is beyond the scope of this manuscript to give a comprehensive understanding of how the signalling from cKIT can impact the BO state, however we feel that the uncovering of links to oestrogen signalling and the observation of a key tight junction protein translocating away from the plasma membrane form the basis for exciting further investigation.

Specific Comments

1. The conclusions remain highly speculative and extend beyond the evidence presented. Although the data support changes in gene expression, pathway activity and protein localisation following KIT knock-out, they do not directly demonstrate that cKIT maintains the Barrett's phenotype or delays/prevents progression to oesophageal adenocarcinoma. Data demonstrating increased transformation. dysplasia and tumour formation are all missing to support this conclusion. These experiments are required to justify the conclusion and even the title. As its stand its misleading

Following on from the above response, it is beyond the scope of the scope of this work to fully demonstrate that cKIT maintains the Barrett's phenotype. This would require a significantly more detailed examination of all the downstream signalling events and their ability to reduce the cancer promotional activity. Only armed with all of the promotional features required to convert Barrett's cells to OAC cells could this be done. For example, the Reviewer suggests that we should demonstrate increased dysplasia from Barret's cells. This would require experiments in tissue and clear, definition of the molecular features of dysplasia. These are currently unknown.

2. The authors state: "Based on previously reported meta-analysis of patient data (Maag et al., 2017, Jones, 2021)we were able to interrogate the role of all known RTKs in BO by comparison with

© 2026. Published by The Company of Biologists under the terms of the Creative Commons Attribution License (https://creativecommons.org/licenses/by/4.0/).

data from normal and OAC tissue. Amongst all the RTKs reported, cKIT showed low overall expression,howevers unlike the other RTKs, showed elevated expression only in HGD Barrett's tissue" It is unclear which data are the author drawing this conclusion from. Clarification is required.

We thank the reviewer for bringing our attention to this. This issue is covered in Supplementary Figure 1 which was generated from the data provided in the Maag et al paper. We have covered the Reviewer's issue as follows: 1) placed an asterisk next to 'KIT' and importantly, 2) explained the abbreviations of the states of the disease in the legend.

3. The evidence supporting the KIT knock out (line 58) at this point is not sufficiently convincing. The authors should repeat the western blot experiment and also provide the complete, uncropped blots as supplementary data to allow assessment of band specificity and data quality.

The western blot suffers from high background signal which was due to the quality of the antibody and the low expression level of cKIT. In Materials and Methods we provide the details for the genetic lesion for the clone, which was determined by Sanger sequencing. The original sequencing files are deposited in the Mendeley repository. This can be accessed as described in the Data Resource Availability section.

4. The authors' state: "This suggests that the deletion of the KIT gene results in a different transcriptional profile which makes cells sensitive to BA treatment." This conclusion is not supported by the data presented. The PCA presented only demonstrates differences in transcriptional responses following BA treatment but does not provide any evidence or suggestion that KIT-KO cells are more sensitive to bile acid exposure. To make such claim appropriate functional assays such as cell viability, proliferation, apoptosis assay is required.

We thank the reviewer for this comment. We would draw attention to the pattern of separation in the PCA (Fig. 3A). CPB cells with and without BA treatment occupy the same region of the plot, whereas $CPB^{cKIT-/-}$ cells with and without BA treatment separate into diagonally opposed quadrants. Were BA to exert an equivalent effect on both lines, comparable displacement would be expected. This asymmetry indicates that loss of cKIT results in a larger transcriptional response to the same insult.

We accept, however, that our original use of the term "sensitive" implied a functional consequence that the transcriptomic data alone cannot establish. We have therefore revised the text to state that deletion of the *KIT* gene alters the baseline transcriptional profile, resulting in the cells being more responsive to BA treatment. The revised passage also sets out the basis for this interpretation more explicitly.

We agree that functional assays such as viability, proliferation or apoptosis would be required to demonstrate a change in sensitivity in the phenotypic sense.

5. With Figure 3E, although differences in gene expression are somewhat apparent, but it lacks statistical analysis. Without appropriate statistical testing, it is difficult to determine whether the observed differences are biologically meaningful.

We are grateful for this comment. The measured levels are low and statistical comparison is not sufficient to make the statement regarding comparative expression In response we have removed the mention of compensatory expression of the ERs and GPER1 in response to BA treatment and cKIT knock out.

6. The Occludin localisation in Figure 4 which appears to be major claim of this paper requires further supportive experiments. Although the immunofluorescence images are consistent with the authors' description. The images are of relatively low magnification making accurate assessment of subcellular localisation difficult. I recommend that the authors validate these findings using subcellular fractionation followed by Western blot analysis for Occludin, together with appropriate nuclear and cytoplasmic marker proteins. Such experiments would provide considerably stronger evidence for the proposed nuclear translocation of Occludin.

© 2026. Published by The Company of Biologists under the terms of the Creative Commons Attribution License (https://creativecommons.org/licenses/by/4.0/).

We thank the reviewer for this suggestion. We would argue that, given the size of the nucleus relative to the resolution of our confocal images, there is sufficient resolution to assess nuclear localisation of Occludin reliably. To illustrate this, we have provided an example of two neighbouring cells, one with and one without nuclear Occludin (below). Fluorescence intensity of both DAPI (marking the nucleus) and Occludin was measured along a line drawn across the two cells. In the first cell, the Occludin signal is low where the DAPI signal peaks, indicating exclusion from the nucleus; in the second, the Occludin and DAPI signals coincide, indicating nuclear localisation.

In addition, because large numbers of cells were counted, we developed a simple ImageJ plugin to assist the manual scoring of cells with nuclear Occludin. The plugin maps the filled outline of each nucleus onto the Occludin channel and extracts the corresponding area, producing either a ring (Occludin excluded from the nucleus) or a filled nucleus (Occludin present in the nucleus). This plugin has been deposited in the Mendeley repository, as detailed in the 'Data Availability' section.

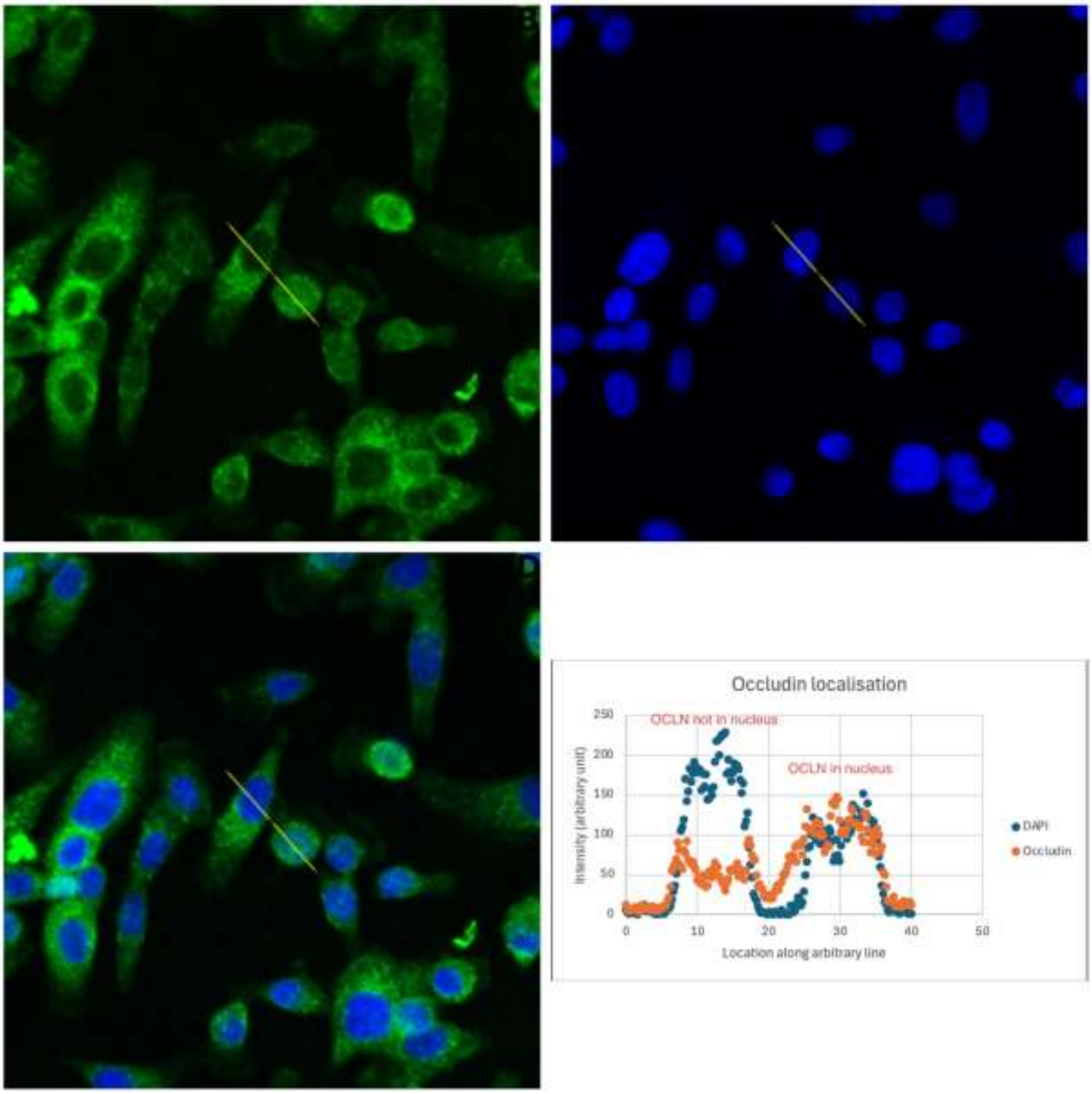

7. I will also recommend that, to strongly validate the role of cKIT, authors should re-express KIT in KIT-KO cells and demonstrate if this restore the lost functions or changes in gene expression observed in KIT Ko cells.

© 2026. Published by The Company of Biologists under the terms of the Creative Commons Attribution License (https://creativecommons.org/licenses/by/4.0/).

We thank the reviewer for this suggestion, which would indeed be an elegant validation of the role of cKIT. However, generating and validating a rescue line, followed by repeat transcriptomic and immunofluorescence analyses across the relevant conditions, represents a substantial body of additional work that falls outside the scope of the present study. We would note that our cKIT inhibitor data provide an independent line of evidence supporting the knockout findings. Although not a rescue experiment, pharmacological inhibition of cKIT recapitulates the effect on Occludin localisation observed in $CPB^{cKIT-/-}$ cells (Figure 4 and Supplemental Figure 4), indicating that the phenotype arises from diminished cKIT activity rather than from an off-target consequence of the knockout. Taken together with the matched comparisons between CPB and $CPB^{cKIT-/-}$ cells, we believe these data establish the association between loss of cKIT activity and the observed changes in gene expression and Occludin localisation.

Reviewer 3:

In this manuscript, the authors investigate the mechanism in the pathogenesis of Barrett's Oesophagus, demonstrating how the receptor tyrosine kinase cKIT cross-talks with and regulates estrogen receptor signaling to stabilize a pre-malignant tissue state. By combining transcriptomic profiling, bioinformatic pathway modeling, and targeted pharmacological assays using bile acid treatments and selective estrogen receptor modulators, the study addresses a highly interesting and clinically relevant hypothesis. The overall experimental design is logically structured and the bioinformatic road mapping is sound; however, the technical execution and data presentation currently fall somewhat short of the standards required to support the authors' central claims. Significant revisions are required to resolve these critical technical flaws before the manuscript's conclusions can be fully verified.

We thank this Reviewer for their positive comments regarding the “highly interesting and clinically relevant hypothesis” as well as the logical structuring and sound bioinformatic road-mapping.

Major Revisions

1. Technical Failure of Knockout Validation and Missing Quantification (Supplemental Figure 3)

* Issue: The Western blot intended to verify the CRISPR-mediated knockout of cKIT in single-cell clones (Supplemental Figure 3) suffers from a fatal biochemical validation flaw. The housekeeping loading control (IB: Tubulin) is profoundly uneven across the gel; it shows a strong band for parental CPB, P1B2, and P1B4, but is severely depleted and nearly invisible in the exact lane for the selected knockout clone (P2B3) and clone P3C2. Furthermore, no densitometric quantification is provided. The blot also appears to be very closely cut.

This is an important comment and one that we have tried experimentally to address. The expression of cKIT in cells is low and the sensitivity of the commercially available antibodies is not ideal. We have attempted to improve the image of the blot and believe that, although the control marker tubulin is not even, there is no evidence for a band corresponding to cKIT to be seen in the selected clone.

* Comment to Author: A uniform loading control is mandatory to conclude that a target band's absence is caused by a genetic knockout rather than an underloaded lane or sample degradation. Because the Tubulin signal drops off precipitously in the P2B3 lane, the apparent absence of the 150 kDa cKIT band cannot be trusted. Since this single clone forms the absolute foundation for every phenotypic essay in Figures 2, 3, and 4, the core conclusions are currently unverified. The authors must repeat this Western blot ensuring equalized total protein loading with robust, uniform Tubulin bands across all lanes, alongside relative densitometric quantification with replicates, or provide definitive Sanger sequencing/ICE genomic confirmation of the out-of-frame knockout.

We thank the reviewer for raising this point. The Reviewer offers Sanger sequencing or genomic confirmation of the out-of-frame knockout as an alternative form of definitive validation, and we are able to provide this. As stated in the Materials and Methods under 'cKIT CRISPR knockout', the P2B3 clone carries a large insertion in exon 3 that introduces a premature stop codon. The Sanger sequencing data confirming this have been deposited in the Mendeley repository, as detailed in the

© 2026. Published by The Company of Biologists under the terms of the Creative Commons Attribution License (https://creativecommons.org/licenses/by/4.0/).

'Data Availability' section, and provide direct genomic evidence of the knockout that is independent of protein detection.

Regarding densitometric quantification, we are unable to provide normalised values for the P2B3 lane because the cKIT signal is zero following background subtraction, and a ratio to Tubulin cannot therefore be calculated. We acknowledge that the blot suffers from high background, reflecting both the properties of the antibody and the low expression of cKIT in CPB cells. The original uncropped blots have been deposited in the Mendeley repository.

We would further note that our cKIT inhibitor data provide independent functional support for these findings. Pharmacological inhibition of cKIT recapitulates the effect on Occludin localisation observed in the P2B3 clone, indicating that this phenotype arises from diminished cKIT activity rather than from a clone-specific artefact.

2. Lack of Statistical Significance and Massive Variance in Target Expression (Figure 3E)
* Issue: The manuscript claims that cKIT knockout explicitly modulates Estrogen Receptor signaling. While the PROGENY pathway inference (Figure 3D) hints at this trend, the actual, direct gene expression levels (FPKM values) shown in Figure 3E tell a contradictory story. The error bars for ESR1 and GPER1 are highly expansive, heavily overlapping between the control CPB, CPB + BA, and CPB cKIT-/- + BA conditions. Shockingly, there are no statistical significance markings (p-values or asterisks) anywhere on this chart.
* Comment to Author: A claim of specific pathway regulation cannot be substantiated when the individual gene expression levels of those target receptors lack statistical significance and exhibit overlapping variances. Please perform an appropriate statistical test (e.g., ANOVA with post-hoc corrections) on the data in Figure 3E and clearly display the resulting significance markers. If the difference is non-significant due to variance, the overarching claim regarding direct estrogen receptor regulation must be heavily scaled back.

This Reviewer has raised a similar point to Reviewer #2. We agree with the conclusions drawn by the Reviewers and have responded by removing reference to comparative expression of the different oestrogen receptors. Clearly, it would be interesting if there was a compensatory expression of GPER1 compared to the ERs in response to BA treatment, however our data cannot confirm this feature

3. Morphological Representation Concerns (Figure 2A)
* Issue/Comment to Author: While the box plots on the right of Figure 2A demonstrate a statistically significant shift in cell area and circularity, the microscopy panel on the left is composed entirely of heavily cropped individual single cells. Presenting singular isolated cells creates an opening for confirmation bias. Please replace these individual crops with wide-field, representative phase-contrast views of the cell monolayer cultures to accurately communicate the uniform phenotypic shifts of the population. Alternatively, the singular isolated crops could be used as zoomed-in blocks placed above the wide-field images.

We thank the reviewer for this comment. The original uncropped images have been deposited in the Mendeley repository, as detailed in the 'Data Availability' section. The individual cells shown are representative of a wider field of cells and of multiple images acquired across independent experiments. Cells were deliberately imaged at low density to allow accurate measurement of cell area and circularity; consequently, the uncropped fields consist largely of empty space and would not provide the confluent monolayer views the reviewer describes. We would also note that the quantification presented on the right of Figure 2A is based on n = 80 cells per line (added to the legend), so the phenotypic shift reported reflects measurements across a large population of cells rather than the selected examples shown.

4. Mismatch in Figure 3 Text, Panel Layout, and Legend
* Issue/Comment to Author: There is a major layout breakdown in Figure 3. The figure features panels A through E. However, the Figure 3 legend skips panel C (the volcano plot) completely in its descriptive lettering sequence and conflates the subsequent panels, incorrectly labeling the PROGENY heatmap as (c) and the Estrogen Receptor expression chart as (d). Please carefully re-align the Figure 3 legend text to perfectly mirror panels A, B, C, D, and E.

© 2026. Published by The Company of Biologists under the terms of the Creative Commons Attribution License (https://creativecommons.org/licenses/by/4.0/).

We thank the Reviewer for pointing out this oversight. We have corrected it.

Minor Revisions

1. Inconsistent Nomenclature and Labeling Conflicts (Figure 4B)
* Issue/Comment to Author: Figure 4B contains conflicting text elements that hamper scannability. The color-coded legend box on the right categorizes the independent variables as an "Inhibitor", while the data matrix underneath the x-axis categorizes them as an "ER modulator". Additionally, the compound names are inconsistently abbreviated between the two spaces (e.g., TAMOX vs Tamoxifen; ISCK vs ISCK03; TAMOX+ISCK vs ISC+Tam). Please choose a single uniform naming convention and apply it to both the legend box and the x-axis data table.
We thank the Reviewer for pointing out this oversight. We have corrected it.

2. Textual and Graphical Errors (Figures 1, 2, and 3)
* Issue/Comment to Author: Please fix the following errors during manuscript revision:
o Figure 1B: The bottom circle of the Venn diagram contains a typo, reading "CPC cv CPD" instead of "CPC vs CPD".

We thank the Reviewer for pointing out this oversight. We have corrected it.

o Figure 2A Legend: The text inside the legend for Figure 2a completely swaps the directional layout of the panel. The cell images are on the left (not the right), and the quantitative boxplots are on the right (not the left). Additionally, the sentence ends abruptly with an unedited fragment ("...staining of."), and falsely claims the cells are stained with crystal violet when they appear to be standard gray phase-contrast images. Please rewrite this legend for accuracy, clarity, and grammatical completeness.

We thank the Reviewer for pointing out this oversight of the panel layout. We have corrected it. We confirm that the cells were stained with crystal violet.

o Results Text: Clean up typos such as "amongst the DEGsS which for BA treated..." to "amongst the DEGs which were treated..."

We have addressed this issue.

Second decision letter

MS ID#: bio.062802R1

MS Title: Regulation of oestrogen signalling by the receptor tyrosine kinase cKIT stabilises the Barrett's Oesophagus pre-malignant state

Authors: Kin Man Suen, Safoura Zahed Mohajerani, Poppy G. Tyrer, Henry W. Clark, Christopher M. Jones and John. E. Ladbury

I am happy to tell you that your manuscript has been accepted for publication in Biology Open, pending our standard publication integrity checks. It was accepted on 3rd August 2026.

© 2026. Published by The Company of Biologists under the terms of the Creative Commons Attribution License (https://creativecommons.org/licenses/by/4.0/).
